# Effect of Preventive Hormonal Therapy on Breast Density: A Systematic Qualitative Review

**DOI:** 10.1155/2014/942386

**Published:** 2014-04-27

**Authors:** Virginie Lienart, Birgit Carly, Xin Kang, Laura Guzy, Anna-Maria Sajovitz, Fabienne Liebens

**Affiliations:** ISALA Breast Unit and Prevention Centre, OB-GYN Department, CHU Saint Pierre, 290 rue Haute, 1000 Brussels, Belgium

## Abstract

Breast density (BD) is recognized as one of the strongest independent risk factors of breast cancer (BC). Unlike most other risk factors, BD can be modified, suggesting that it may be a biomarker for preventive interventions. We conducted a qualitative systematic review to address the effect of preventive hormonal therapy on BD. Among the 26 relevant studies, 10 assessed the effect of tamoxifen on BD (TAM: *n* = 2 877), 9 that of raloxifene (RLX: *n* = 1 544), and 7 that of aromatase inhibitors (AI: *n* = 416). The studies were characterized by a large heterogeneity in designs and in methods of BD measurement. BD could be reduced by TAM (10 studies/10). However, the effect of RLX and AI on BD remains unclear due to conflicting results between studies. Consequently, it is crucial to develop practical, accurate, and reproducible methods of measurement in order to be able to compare the effect of preventive hormonal agents on BD and to determine whether change in BD can be used as a predictor of response to therapy.

## 1. Introduction

Breast density (BD) is that proportion of breast occupied by radiological dense tissue reflecting breast tissue composition. Dense areas represent fibroglandular tissue when nondense areas correspond to fatty tissue [1]. BD is recognized as one of the strongest independent risk factors of breast cancer (BC) apart from age and genetic mutations [2, 3]. Women in the highest categories of BD have a 4- to 6-fold increased BC risk compared to women in the lowest categories [4]. The association of BD and BC risk is present in all ages and is not an artifact of masking bias [5]. Although aging and overweight are risk factors of BC, BD is negatively correlated with age as well as with body mass index (BMI) [6]. To explain this paradox, it has been hypothesized that BD reflects the cumulative exposure to factors that stimulate growth of breast cells since puberty and influence BC incidence [7–9]. Details on available methods of BD measurement have been extensively described including qualitative, semiquantitative, and quantitative computerized, fully or not, automated methods [10, 11]. The first visual classification of the appearance of the breast was described by Wolfe in four categories: N1, P1, P2, and DY with density increasing from N1 to DY [1]. The most widely used qualitative classification is the BI-RADs system developed by the American College of Radiology in four descriptive categories: (1) almost entirely fatty, (2) scattered fibroglandular tissue, (3) heterogeneously dense, and (4) extremely dense. The new (fourth edition) BI-RADs involves combined qualitative and quantitative assessments with corresponding quartile of dense areas on the film from <25% to >75% [12]. In the last decade, more studies have been conducted with computer-assisted techniques using digitized copies of the mammogram, full digital mammography, and more recently, magnetic resonance imaging (MRI) in order to obtain more objective assessment. Despite these recent inputs, nowadays it remains unclear whether BD is best expressed in terms of absolute dense area or percentage dense area [10]. Although the mechanisms by which BD affects BC risk are not well understood, an estimated 16% of all BC have been attributed to BD higher than 50% [2]. Unlike most other risk factors for BC, BD can be modified, suggesting that it may be a biomarker for preventive interventions [13]. Postmenopausal hormonal therapy (HT) with combined estrogen and progesterone has been shown to increase BC risk and BD. Recently, it has been suggested that the risk of BC and advanced disease is higher among postmenopausal HT users when they have high BD [14]. Since postmenopausal HT may increase BD, one may also wonder to what extent preventive hormonal agents could reduce BD. Furthermore it has been recently shown that the 12- to 18-month change in BD could be a predictor of response to tamoxifen in the preventive setting suggesting that reducing BD may translate into decreased BC risk [13]. Two groups of hormonal agents have proven efficacy in reducing BC risk in large prospective randomized trials. These include selective oestrogen receptor modulators (tamoxifen, raloxifene) and aromatase inhibitors (AI) (exemestane) [15]. Tamoxifen and raloxifene have been approved by the Food and Drug Administration for reducing BC risk but not by the European Medicines Agency. This paper reviews systematically available data concerning the influence of preventive hormonal therapy on BD.

## 2. Material and Methods

Using online databases (Medline, PubMed, Cancerlit, Cochrane Controlled Trials Register, and Google), we conducted searches to identify all published reports dealing with changes in BD associated with preventive hormonal therapy. Since different patterns of BD were identified by Wolfe in 1976, we looked for articles published between 1976 and 2012 [1].

Preventive agents included in this review were tamoxifen (TAM), raloxifene (RLX), and exemestane (EXM) [15]. Results on the effects of anastrozole (ANAS) in the preventive setting are not yet available. However, since ANAS and letrozole (LET) have shown a stronger reduction in the risk of contralateral tumours than Tam in the adjuvant setting, we also included both agents in our review [16, 17]. The search strategy included in various combinations the following keywords: “name of the preventive hormonal agent and* breast density, mammographic breast density, mammography, MRI, dense breast, breast cancer risk, prevention, hormonal therapy…*.”  If reports identified according to these criteria referred to other papers not identified in the initial search, these were also reviewed when relevant to the main questions. The search was complemented by consulting review articles and BC conference proceedings. All available abstracts were reviewed and the full text of an article was consulted by a different reader (FL) when eligibility was ambiguous.

To be included in this review, studies had to be written in English or French, to provide details on the methods used to determine BD either by mammography (screen analog film and/or digitized) or MRI and to give information on at least one of the following confounding factors: age, BMI, menopausal status, family history, HRT use, previous benign breast biopsy, age at menarche, and age at first birth. Since BD may be influenced by radiotherapy and chemotherapy [18–20], studies in BC patients providing no data on BC treatment were excluded.

We also excluded case reports and review studies. Data were extracted from the manuscripts using a standardized methodology and according to the checklist of the PRISMA guidelines [21–23].

Quality assessment was performed on a data extraction form developed for the review and based on previously reported method [21]. The following trial “quality characteristics” was assessed:

1. design: appropriateness of the design to evaluate the endpoint (coded as “adequate”, “unclear” or “inadequate”);
2. patients selection and eligibility: clear eligibility criteria mentioned and similar baseline characteristics between groups (coded as “done,” “partly done,” and “not done”);
3. missing data: that is, missing mammograms (coded as “yes,” “no,” “unclear”);
4. compliance: report of the measure to which participants complied with taking the preventive hormonal agents (coded as “done” and “not done”);
5. outcome assessor blinding: that is, whether the mammogram reader was blinded to the intervention (coded as “yes,” “no,” “unclear”);
6. reproducibility: data provided on the reliability of the BD measurement at the different time sequence of the treatment (coded as “done,” “partly done,” and “not done”);
7. duration: appropriateness of the duration of the intervention to assess outcome (coded as “yes,” “no,” “unclear”);
8. confounding factors: appropriate testing of major factors that could interact with treatment effect: BD at baseline, age, menopause status, and BMI (coded as “done,” “partly done,” and “not done”).

These quality criteria were independently assessed by all investigators except one (CB). For each criterion we obtained five evaluations. Based on these evaluations, we calculated the number of studies that met appropriately each quality criterion.

**Figure 1:**
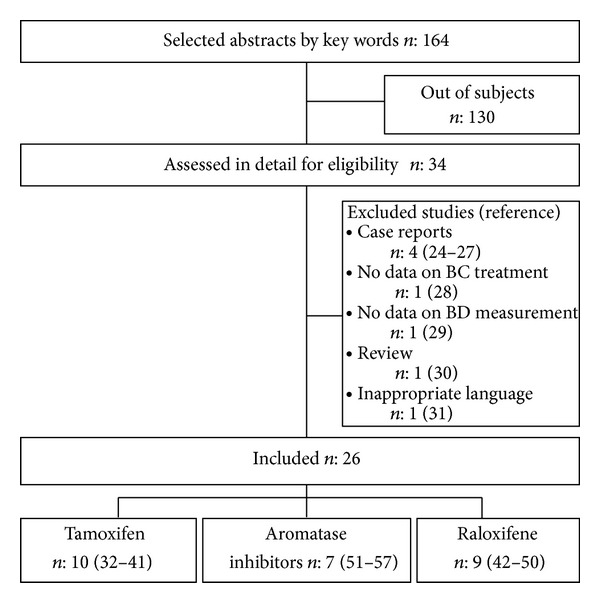
Flowchart illustrating movement of papers from search to inclusion.

We retrieved 164 abstracts using the keywords; 130 were found to be irrelevant to the topic and 34 full-text papers were selected from the remaining abstracts (Figure 1). We then excluded 4 case reports studies [24–27], one study with no data on BC treatment [28], one study with no information on methods of BD measurement [29], one review study [30], and one study with full text in unselected language [31]. We retrieved 26 relevant studies. Among them, 10 studies assessed the effect of Tam [32–41], 9 that of RLX [42–50], and 7 that of AI [51–57] on BD (Figure 1). We evaluated the methodology, the characteristics of the studied populations, confounding factors, and the results of these 26 remaining studies. The details of study design were submitted in June 2012 to the Prospero database and registered under the number* CRD42012002536*.

## 3. Results

### 3.1. Risk of Bias

Design was classified as appropriated in 27% of the reviewed studies. Clear eligibility criteria and similar baseline characteristics between groups were provided in 40%. Data on missing mammograms were detailed in 30% of the studies. Compliance with medication was reported in 17%. A clear and realistic attempt to mask the mammogram reader about the treatment was detailed in 58%. Data on the reliability of the BD measurement were mentioned in 17%. Study duration was quoted as adequate in 44%. Finally, description of confounding factors was adequately assessed in 10% of the reviewed trials.

### 3.2. Tamoxifen: Populations and Design (Table 1)

Among the ten studies having assessed the effect of Tam on BD, we found one randomized trial (RT) [33] and two post hoc studies of a subset of mammograms from patients included in RT [32, 35] (Table 1). One study was a nested case control analysis from a prospective RT [41]. Five studies were retrospective, among them three were matched with controls [36, 37, 39]. Finally, since one study retrospectively selected patients from another protocol to form a control group, we classify this study as retrospective [34].

The number of women included in these ten studies ranged from 16 to 1065 (total = 2.877). Four studies included women at high risk of developing BC, based on family history of BC or proliferative benign breast disease diagnosis (ductal carcinoma in situ, lobular carcinoma in situ, or atypical hyperplasia) or a Gail 5-year risk for BC higher or equal to 1.7% [34, 35] or 1.3% [33]. In one of these studies, patients who developed small invasive BC within a period of 3 years before randomization were also included [33]. One additional nested case-control study within a randomized prevention trial of Tam versus placebo (IBIS 1) aimed to investigate the relationship between change in BD under treatment and known BC risk factors. This trial included women diagnosed with BC (cases), but no matching occurred for the control subjects [41].

The five remaining studies evaluated BC patients who received Tam alone or Tam combined with chemotherapy and/or radiation therapy after surgery. Among them, two were case control studies with various control groups [37, 39]. None of these two studies provided data on whether BC patients were matched for treatment such as chemotherapy and/or radiation therapy or not (Table 1). The mean age ranged from 43 to 67 years and in four studies, patients were mainly younger than 50 years of age. Most studies (9 out of 10) included some postmenopausal patients, but none explained clearly the criteria used to define menopause. The total duration of the Tam studies ranged from 1.5 to 6 years.

### 3.3. Tamoxifen: Methods of BD Measurement (Table 1)

Most studies evaluated change in BD under Tam treatment using visual qualitative or semiquantitative methods (*n* = 7, Table 1). In three studies, the authors described various visual methods to score BD on analog mammograms [35, 37, 38]. Only four studies mentioned data on missing mammograms at baseline ranging from 10% to 45% [32, 33, 35, 41].

In six out of eight studies assessing BD in BC patients or in patients who had undergone biopsy for high risk lesions, the unaffected breast free of any surgical intervention was selected for BD measurement [34, 36, 37, 39–41], but information on the side of breast assessed was not available in the two remaining studies. BD was determined in nine studies either on screen film (*n* = 6) or on digitized mammograms (*n* = 3) (Table 1). Six out of these nine studies provided information on incidence used: craniocaudal (CC: *n* = 2) [34, 39]; mediolateral oblique (MLO: *n* = 2) [37, 41]; both (*n* = 2) [35, 38]. Data on BD at baseline were provided in various manners: the proportion of the total breast area that was composed of dense tissue in percent (*n*: 5) [32–35, 41], the BI-RADs or Wolfe classification (*n* = 3) [36, 37, 39], a ratio dense/fat (*n* = 1) [38], and MRI volumetric changes (*n* = 1) [40]. When available, BD at baseline ranged from 31.9% to 60.5% (Table 1). BD was determined by one reader in three studies [33, 36, 41] and by at least two readers in six [32, 34, 35, 37–39]. Three authors provided no information on whether readers were blinded or not to the trial information. In four studies, readers were blinded to treatment arm [32, 33, 39, 41]. Among them, only one mentioned blinding for time sequence of the mammograms [33]. Finally, in two studies, the authors mentioned that “the readers did not have knowledge of the outcome of the first review” [34, 35]. The reproducibility of BD measurement was available in seven studies using various endpoints (Table 1). These endpoints include: inter-observer agreement (correlation between different observers) in two studies [32, 41]; intra-observer agreement (correlation between different readings of the same observer) in two studies [35, 36], and agreement between methods of BD measurement in one [39]. One study provided reproducibility data according to the method of BD measurement. For the computer-aided calculation, reproducibility was determined as the differences between the rescored and original measurements [34]. Finally, one study [38] mentioned that parallelism in the findings of the two radiologists occurred in 24 out of 27 cases. In four out of seven studies, reproducibility was determined only in a subset of mammograms [32, 34, 36, 41].

### 3.4. Tamoxifen: Effects on BD (Table 4)

Most studies showed that Tam reduced BD significantly. Four studies showed a statistically higher decrease in percentage of BD in the TAM group as compared with placebo or control groups: −13.7% at 54 months [32], −19.6% at 24 months [33], −4.3% estimated reduction per year [34], and −9.4% at 62 months [35] (Table 4). The number of women experiencing a decrease in BD was determined in five studies after different treatment durations and ranged from 20.59% to 59.8%. Finally, using MRI technology in a cohort study, the authors found a significant absolute reduction of −5.8% ± 3.8% in %BD after a mean duration of 17.5 ± 5.7 months [40].

Data on some confounding factors of BD at baseline were mentioned in five studies (Table 4) [32–35, 41]. Among them, BMI was negatively correlated with BD at baseline in two studies [32, 33], and young age was more frequently associated with higher BD in four studies [32–35], although, not statistically significant in two [34, 35]. Premenopause, low predicted familial risk, and never smoking status were significantly associated with higher BD at entry in one study [32]. History of past breast biopsy was associated with higher baseline BD in two studies [32, 41].

Some potential confounding factors that could interact with TAM effect were detailed in seven studies in which the authors attempted to evaluate the reduction in BD in different subgroups [32–35, 37, 39, 41] (Table 4). In multivariable analysis, greater reductions in BD were significantly associated with higher BD at entry [32], BMI < 25 kg/m^2^ or less [32], stopping HRT during the study [32, 41], smoking during the study [32], young age [32, 39], past breast biopsy [41], and premenopausal status [32, 37].

### 3.5. Raloxifene: Populations and Design (Table 2)

Among the nine studies having assessed the effect of RLX on BD, we found three RT [42, 43, 49] and three studies that were either post hoc analyses [46, 47] or a substudy [45] performed within RT. Two studies were retrospective without control groups [44, 50], and finally one study was prospective controlled [48] (Table 2). Most of the reviewed studies (8/9) were performed in postmenopausal women. Four out of these eight studies evaluated women with osteopenia and/or osteoporosis [42, 46, 48, 50]. Among them, one study prospectively enrolled women with osteoporosis who received RLX whereas women with osteopenia were enrolled as controls and matched for age at entry and age at menopause [48]. One additional study included women declared to be at risk of osteoporosis and cardiovascular diseases but details regarding these risk factors were missing [49]. Only one study included premenopausal women at high risk of developing BC, based on family history of BC or proliferative benign breast disease diagnosis or a Gail 5-year risk higher or equal to 1.7% [44] (Table 2).

The nine RLX studies totalized 1 544 patients ranging from 37 to 442. The mean age (±SD) of postmenopausal women varied between 50.4 ± 3.6 years and 66.9 ± 5.3 years. In six out of these nine studies, the effect of RLX on BD was compared with the effect of various regimens of hormone replacement therapy (HRT) including combined estrogen progesterone hormone therapy at different doses (cHRT) [42], estrogen only therapy [45, 47], tibolone or cHRT [43], tibolone alone [49], and bazedoxifene [46] (Table 2). Information regarding the menopause definition was provided in five out of eight trials including (1) time since last menstrual period ranging from 6 months to 5 years [42, 43, 46] and (2) serum levels of follicle stimulating hormone and/or estradiol [48, 49]. In one additional study the authors included some patients based on surgical menopause but provided no details on how they confirmed the menopause status of the patients who had not undergone hysterectomy [50]. The interval between HRT discontinuation and study inclusion was mentioned in six out of eight studies [42, 43, 45, 46, 49, 50] and ranged from 1 months to 24 months. The duration of the RLX studies ranged from 3 months to 36 months (Table 2).

### 3.6. Raloxifene: Methods of BD Measurement (Table 2)

In three out of nine studies, calculation of BD was performed using visual qualitative methods alone [42, 49, 50] (Table 2). Computer-assisted segmentation of digitized mammograms using an interactive thresholding software was utilized in other three studies [45, 46, 48]. Two authors described personal methods of BD measurement based on (1) the relative volume of dense tissue [43] and (2) an estrogen-specific heterogeneity radiograph score [47]. Finally, in the remaining study, semiautomated calculation of change in breast volume based on MRI technology was used in association with an interactive thresholding software aimed at determining percent BD [44]. Five authors mentioned information on missing or not technically acceptable mammograms at study entry [42, 44–47] ranging from 0.45% to 48.6%. The number of mammograms readers was available in six out of nine studies and ranged from one to three. Readers were blinded to treatment arm in four studies [42, 45, 47, 49] and both to treatment arm and to time sequence only in one study [46]. Four authors published some data on reproducibility of BD calculations using different endpoints: interobserver reliability at baseline (weighted kappa *r* score: range 0.57–0.70) and after 12 months (range 0.51–0.66) [42], interradiologist correlation at each year of assessment for 3 years and one year after cessation (range 0.63 for 1 year assessment to 0.39 for post treatment calculation) [44], intra-observer variability between baseline and 2-year assessment (range 0.70–0.86) [47], and finally one author mentioned that, in case of discrepancy (9,2–13%), films were reevaluated by the 2 radiologists together for consensus [49]. Seven studies provided information on mammography incidence used: MLO alone [43, 47], CC alone [44, 46], and both [45, 48, 49]. When available, the BD at baseline in postmenopausal [45–47] and premenopausal women [44] ranged from 8.1% to 27.6% and from 7% to 78%, respectively.

### 3.7. Raloxifene: Effects on BD (Table 5)

Two out of nine studies showed that RLX significantly decreased BD (Table 5). Using MRI technology in high risk premenopausal women, the median relative MRI volume (MRIV) decreased after 1 year and 2 years by 17% (95% CI, −28 to −9; *P* = 0.0017) and 16% (95% CI, −31 to −14; *P* = 0.0004), respectively [44]. In postmenopausal women using a computer-assisted evaluation, the image mean index (IMI) decreased significantly by 1.9% (*P* < 0.5) after 2 years of RLX therapy [48]. In the seven other studies, no significant modification of BD was observed. Only two studies provided some confounding factors of BD at baseline with discordant results: one study found that BMI was negatively correlated with BD at baseline [47] while this was not the case in the other study [44]. Only one study looked at therapy-by-subgroup interactions and found no statistically significant interaction for BMI, age at entry, menopausal status, use of HRT, baseline BD, and smoking status [45].

### 3.8. Aromatase Inhibitors: Populations and Design (Table 3)

We found seven studies assessing the effect of AI on BD (LET: *n* = 5, ANAS: *n* = 1, and EXE: *n* = 1). Among these seven studies, two were RT [51, 57] and one was a subgroup analysis of a subset of mammograms from patients included in an RT [52]. Three were prospective single arm trials [53, 54, 56]. The remaining study was retrospective and case controlled [55]. The number of women included ranged from 16 to 104 (total = *n*: 416).

Two studies included women at high risk of developing BC based on history of proliferative benign breast disease diagnosis, proven BRCA 1/2 mutation, or a Gail 5-year risk higher or equal to 1.67% [54] or to 8% [53]. In one of these studies, high risk women received HRT [54]. Three studies addressed the issue of BD in BC patients receiving LET or ANAS. Among them, BC patients were selected if they had completed 5 years of TAM therapy [52] or if they were receiving an AI as their only adjuvant systemic therapy [56] or if they had an estimated baseline BD of at least 25% [51]. The two remaining studies determined BD in postmenopausal women receiving EXE [57] or in postmenopausal women receiving HRT alone compared to women receiving HRT + LET [55]. In two out of four controlled studies, the treatment arm and the control group were not perfectly balanced in regard to age, history of benign breast disease, and family history of BC [51] and BMI, number of tumors, and node positive disease [52]. The median age of the selected patients in the seven studies ranged from 50 years (39–68) to 64.6 years (30–84). Most studies (6 out of 7) included solely menopausal patients with various menopause definitions according to patients' age. For younger women (less than 50 years of age [53, 57] or less than 55 years of age [51]), menopause was defined as no spontaneous menses for at least 12 months and FSH levels in the menopausal range. Two additional studies included patients with hysterectomy and/or bilateral oophorectomy or radiation castration with more than 6 months of amenorrhea [54, 56]. Finally, in one study, patients were declared in menopause based on an accepted age for menopause but no additional criteria [55]. Only one author provided data regarding time since menopause [55]. The total duration of the reviewed studies ranged from 6 to 24 months (Table 3).

### 3.9. Aromatase Inhibitors: Methods of BD Measurement (Table 3)

Four studies evaluated change in BD on digitized mammograms using a computer-assisted thresholding program [52–54, 56]. In three studies, different computer-assisted methods were associated with visual qualitative or semiquantitative measurements based on BI-RADs and/or Boyd classifications [51, 55, 57]. Data on missing mammograms were provided in five studies [51, 53, 55–57] and ranged from 7.4% to 28.5%. In all studies assessing BD in BC patients, the unaffected breast was selected for BD measurement [51, 52, 56]. The CC incidence was used to determine BD in all studies. Data on BD at baseline were provided in various manners. Some used the proportion of dense tissue expressed in percent (*n*: 5 [51, 53, 54, 56, 57]), the Boyd classification scale (*n*: 1 [52]), and the total integrated pixel intensity (IPI) (*n*: 1 [55]). When available, BD at baseline ranged from 13.4% to 40% (Table 3). BD was determined by one reader in all studies (Table 3). Most of the AI studies (6/7) provided information on blinding. In two single arm prospective studies, readers were blinded to time sequence of the mammograms [54, 56]. In the remaining studies, readers were declared blinded to time sequence and patients' treatment [51, 53, 55, 57]. Concerning reproducibility of BD measurement, only one author detailed intra-observer reproducibility for qualitative BI-RADs assessment [55] (Table 3). For computer-assisted methods only one study provided information on the reproducibility of this analysis and mentioned an intra-observer variation not greater than 10% [52].

### 3.10. Aromatase Inhibitors: Effects on BD (Table 5)

Two studies using different methodologies showed that AI significantly decreased BD. Based on a computer-assisted method, one author showed that a statistically significant reduction in integrated pixel index (IPI) occurred in the women who received HT plus LET, whereas no significant change was observed in the women receiving HT alone [55]. In another study using LET, eight out of 16 patients exhibited decreased BD at six months, and eleven out of 16 at 12 months [53] (Table 5). No data on confounding factors of BD at baseline were mentioned in any AI studies. Potential confounding factors that could interact with treatment effect were detailed in four studies [51, 52, 56, 57] (Table 5). The authors found no statistically significant therapy-by-subgroup interactions for BMI and age at entry to the trials.

## 4. Discussion

There is considerable evidence establishing a clear relationship between BD and risk of BC [2, 5, 58–60]. It has been estimated that an increment of 2% in BC risk exists for each percentage increase in BD [61]. In addition, strong scientific data suggest that TAM, RLX, and EXE are reasonable options for reducing BC risk in women who are at increased risk for developing the disease [15]. Despite all these available data, we found few RT (*n*: 6) assessing the effect of preventive hormonal agents on BD. Most studies (*n*: 20) had some collection and selection biases. In addition, there was an important heterogeneity between studies with respect to different confounding factors, selected population, number of patients, missing mammograms, methods and reproducibility of BD measurement, and duration of study. Despite this, we found in 19 controlled studies that TAM reduced BD in all studies (8 out of 8), RLX in one out of seven, and AI in one out of four. Several explanations may account for this discrepancy.

First, the magnitude of BD reduction and the reliability of the assessment may depend on the method of measurement [62]. For instance, Chow et al. [34] found a significant decrease in density in women at high risk of BC who received TAM, measuring BD with a semiquantitative method but not using Wolfe or BI-RADs classification. The only study relating BD decrease to RLX assessed volumetric BD [63] by automated technique in full-field digital mammograms [43], while the other RLX studies used different methods of BD assessment. Mousa et al. [55] observed, when measuring BD by quantitative image analysis software, a significant reduction in women who received HT plus LET as compared to HT alone. This was not the case when BD was visually analyzed by radiologists. Nielsen et al. [47] determined BD changes via BI-RADs and E2-specific heterogeneity scoring; the latter aims to quantify a specific biological effect on mammographic patterns. They found that RLX modified the E2-specific heterogeneity score, but not BD assessed using BI-RADs. Similarly, Eng-Wong et al. [44] studied the effects of RLX on BD calculated by a semiquantitative thresholding technique and MRI-breast volume (MRIV). They found no significant change in BD calculated on mammograms whereas MRIV decreased during RLX treatment. These results suggest that determination of MRIV changes offers a more reproducible and sensitive measure of fibroglandular tissue [64]. Decensi et al. [33] observed that, at baseline, women with digital measurement had a BD that was nearly 16% lower compared to those with analog-film screen. In this study, BD was determined on digitized mammograms using an interactive threshold method (Cumulus software [61, 65]). It has been suggested that density measured with this software is a better predictor of risk than density assessed visually [5] and has an established sensitivity of 5%, allowing the detection of relatively small changes in BD [66, 67]. Nevertheless, the evaluation of BD based on mammogram entails major problems: tissue overlapping, positioning difference of the breast, variation in the degree of compression as well as calibration of mammography units, and changes in exposure factors and doses used. These represent additional limitations when using mammograms to assess BD changes over time [68, 69]. Although controversial, the incidence use to determine BD may also play a role as it has been shown that density estimates on the CC tend to be higher than on the MLO [62, 70, 71]. Other potential factors that could have confounded the accuracy of BD measurements include the huge heterogeneity of mammogram incidence used, the lack of systematic evaluation of intra-interreaders reproducibility, and the fact that mammogram readers were not always blinded to time sequence of treatment.

Second, the discrepancy in our results may also be explained by differences in selected populations. The greatest decline in BD typically occurs around 45 years of age and plateaus at approximately 60 years of age [72]. In agreement with this, Cuzick et al. [32] found a significant interaction of treatment effect with age. A minimal decrease in BD was observed for women over 55 years of age treated with TAM (1%), compared to women younger than 45 years of age (13%). Similar results were observed by Meggiorini et al. [39]. Tam studies included larger numbers of patients, both pre- and postmenopausal, who were also at increased risk of BC, whereas the RLX and AI studies mostly included small numbers of mainly postmenopausal patients. This heterogeneity in the age of the populations is underlined by the variety of baseline BD: in the Tam studies, the RLX studies, and in the AI studies, baseline BD ranged from 31.9% to 60.5%, from 8.1% to 27.6%, and from 13.4% to 40%, respectively. This difference raises the question that perhaps the BD in the RLX and AI treated women may not have been elevated enough to detect a significant change. In addition, menopause is thought to have a more important influence than age on the decline in BD [72–74]. Although a standardized definition of menopause is frequently missing, the influence of menopause on BD changes is confirmed in several reviewed studies. Hong and Ki [37] found in BC patients that 87% of premenopausal women with BC had a decrease in BD with Tam use, whereas only 29% of postmenopausal women experienced a decrease. A similar trend was observed in the Brisson's study [35], performed on women with high risk for BC. In a retrospective analysis comparing the effects of bazedoxifene and RLX on BD in postmenopausal women with osteoporosis, Harvey et al. [46] observed that neither significantly decreased BD. In contrast Lasco et al. [48] observed a reduction of BD using RLX in a population of postmenopausal women. However, it should be noted that women enrolled in the Lasco's study were younger (mean age: 52 yrs) than those evaluated in the Harvey's study (mean age: 59 yrs). In addition it is well documented that weight gain is common among women diagnosed with BC [75] and among postmenopausal women [76]; as weight increases, breasts tend to become more lucent. Unfortunately, most of the RLX and IA reviewed studies were not sufficiently powered to properly assess the interaction of BMI with treatment effect.

Third, the discordant results may also be attributed to different biological effects of selected preventive agents. Selective estrogen receptor modulators (SERMs: Tam and RLX) are nonsteroidal compounds that elicit estrogen agonist effects in some tissues, such as bone and the cardiovascular system, and estrogen antagonist effects in others, such as the breast. The tissue specificity of SERMs may be related to the existence of (at least) two different isoforms of the estrogen receptor with distinct signaling properties [77, 78]. On the other hand, the AI reduce breast and circulating estrogen levels in postmenopausal women by blocking the conversion of androstenedione to estrone and testosterone to estradiol by cytochrome P450 (CYP) 19, aromatase [79]. In addition, growing evidence shows that BD reflects the degree of stromal and epithelial proliferation and may be closely linked, mostly in premenopausal women, to some growth factor activity such as insulin growth factor (IGF)-1 [11, 80–82]. Furthermore, a recent study showed that aromatase immunoreactivity is increased in dense breast tissue and that stromal cells from dense regions have higher levels of aromatase expression than epithelium [83]. However, studies correlating BD with serum estrogen levels have been inconsistent with most studies supporting either no association or an inverse association with estrone or estradiol levels [11]. In this review only four studies evaluated whether preventive agents' effects on BD could be mediated by different biological mechanisms. Two SERMs studies addressed the IGF-1 pathway: Decensi et al. [33] showed that, during the 2-year intervention, Tam significantly lowered IGF-I and BD by 12% and 20%, respectively. Lasco et al. [48] observed that long-term treatment with RLX is able to reduce BD. In women treated with RLX, there was a negative correlation between IGF-1/IGFBP-3 ratio and BD. Although these data confirm previous studies showing that SERMs could decrease IGF-1 and increased IGFBP-3 plasma levels, hence reducing the IGF-1/IGFBP-3 ratio [84–86], they could not clarify the link between BD changes and IGF-1 pathway. Conversely, Cigler et al. [51] noted a significant increase in serum IGF-1 levels in the LET group compared to the placebo group. Whereas Fabian et al. [54] found no change in IGF-1 and IGF-1/IGFBP-3 ratio in women receiving LET. Interestingly, it has been recently shown that Tam interacts with the mammary stroma and that these interactions dictate epithelial cell function. Given that BD also is influenced by stromal tissue, this finding suggests a specific effect of Tam on BD [87].

Finally, individual polymorphisms in drug metabolizing enzymes such as CYP2D6 (Tam metabolizing genes) and CYP19 (aromatase genes) may play a role in sensitivity or resistance to preventive therapy [88, 89]. All these data underscore potential different mechanisms of action between SERMs and AI on BD but need further confirmation.

## 5. Conclusion

In the event that preventive hormonal agents protect women from BC by inducing changes in BD, it may be important to identify women in whom BD decreases and who will benefit from these therapies. In this review, we found that BD could be reduced by TAM. However, the effect of RLX and AI on BD remains unclear due to conflicting results between studies. These differing results highlight numerous biases associated with the design and conduct of trials that assessed BD as endpoint. A major one is the variability of methods of BD measurement retrieved. Consequently, it is crucial to develop practical, accurate, and reproducible methods of measurement in order to determine whether BD can be used as a predictor of response to therapy. Moreover, as new therapies become available in the preventive setting for women at high risk of BC, our results support the need for further larger and well conducted studies aimed to understand whether specific characteristics predict BD changes in response to such therapies.

## 6. Implications for Practice

Tam could reduce BD. This reduction is higher in young and premenopausal women. Changes in BD may be a marker of response to Tam therapy [41]. Although the clinical relevance of this potential relationship merits further investigations, there are no immediate implications for practice. No definitive results could be found for RLX and AI.

## 7. Implications for Research

In the absence of sufficient data on the effects of RLX and AI on BD in women at high risk of BC, it would be helpful to assess these agents in a large, powered and high quality RT. This trial should last at least 2 years, assess BD with a reproducible and reader-blinded method, and determine if some treatment effect is influenced by other BC risk factors such as age, BMI, family history, past breast biopsy, age at menopause, age at first live birth and use of HRT.

## Figures and Tables

**Table 1:** Tamoxifen (TAM): characteristics of the reviewed studies (*n*: 10).

Authors/year	Endpoint	Design	High risk definition	Population/number of cases	Age	Method of BD measurement/time	Mammograms readers	Reproducibility	BD baseline
Cuzick et al. 2004 [32]	Effect of tamoxifen on breast density	Subgroup retrospective analysis within IBIS 1 randomized double-blind study	Family history of BC or proliferative benign breast disease	7152 healthy women at high risk of BC/818 BC—free women (388 in the **TAM** group and 430 in the **placebo** group)	35–70 y	Visually according to the Boyd classification scale/at baseline and during 54 months of treatment, at 12- to 18-month intervals	A single consultant radiologist blinded for treatment group + specially trained research nurse	In a subset of mammograms from 70 women/correlation coefficients: 0.91 (baseline readings), 0.88 (18 months), and 0.74 (54 months)	**Placebo**: 42.6% (95% CI: 39.6%–45.6%);** TAM**: 41.9% (95% CI: 38.8%–45.0%)

Decensi et al. 2009 [33]	Effect of tamoxifen^1^ and fenretinide (alone and in combination) on IGF-1 and breast density	Randomized, double-blind, placebo-controlled trial with a 2 × 2 factorial design	Intraepithelial neoplasia or small invasive BC in the 3 yrs before random assignment or a Gail 5-year risk for BC ≥ 1.3%	880 premenopausal women at high risk of BC/235 (**TAM **alone: 55; **fenretinide **alone: 56; **combination**: 59; placebo: 57)	**TAM alone**: 46.2 y ± 5.0 (range 32–57),** fenretinide alone**: 46.2 y ± 5.2 (range 30–56), **combination**: 46.9 y ± 4.5 (range 38–54),** placebo**: 46.5 y ± 4.3 (range 36–54)	Visually according to the Boyd classification scale and computer-assisted calculation/at baseline and at 12 and 24 months on analog screen films or digital scans	Single trained radiologist, blinded as to time sequence and treatment arm	NA	**IEN/T1** ^ 2^: 46.6% (SD ± 16.4%); **Gail**: 49.5% (SD ± 15.2%)^3^

Chow et al. 2000 [34]	Effect of tamoxifen on breast density	Secondary analysis of a prospective safety and feasibility study	Diagnosis of DCIS, LCIS, or atypical hyperplasia or a Gail 5-year risk for BC ≥ 1.7%	32 women at high risk of BC/**TAM** group: 28 patients and **control **group: 20 patients from another protocol	**TAM**: mean 49.5 y (range 36–74 y) and controls: mean 51 y (range 42–57 y)	4 methods: (a) Wolfe and BIRADs; (b) Boyd classification scale; (c) computer aided calculation from digitized mammograms/4 days to 9 months (mean 2.3 months) before the start of TAM therapy and annual mammograms during 2 y	Two independent readers for methods (a) and (b); single reader for method (c)	High interobserver variability for (a) and (b). For (c): in a subset: differences between second set and first set: mean −2.2 ± 6.9% (range −15.5% to 11.0%).	Initial digital scores: **TAM**: 31.9% (SD ± 19.0%) and controls: 29.7% (SD ± 12.0%)

Brisson et al. 2000 [35]	Effect of tamoxifen on breast density	Subgroup analysis within NSABP P1 randomized double-blind study	Diagnosis of LCIS or a Gail 5-year risk for BC ≥ 1.7%	184 healthy women at high risk of BC/69 (**TAM** group: 36; placebo: 33)	**TAM**: mean 50.9 ± 8.0 y and** placebo**: mean 50.3 ± 6.4 y	Visually according to Wolfe's parenchymal pattern and a 22-score percentage of the breast showing densities/pre- and posttreatment mammograms	Two independent blinded readers (third reading if necessary)	Weighted kappa comparing assessments (pre/posttreatment): 0.82/0.83. Intraobserver correlation coefficients: 0.92/0.89	Mean BD at randomization: **TAM**: 60.3% ± 18.5% and **placebo**: 60.5% ± 24.2%

Atkinson et al. 1999 [36]	Effect of tamoxifen on breast density	Retrospective case control study, 1 : 2 matching by age and menopausal status	BC	282 (**cases**: 94 BC patients who had received **TAM** and **control **group: 188 age matched cancer free women)	Mean 59 ± 3.3 y (range 50–64 y)	Visually according to Wolfe's parenchymal pattern/at baseline and at 19.6 months (cases) and 14.3 months (controls)	A single reader not blinded	For a subset (13%) of mammograms intraobserver agreement. Correlation coefficient: 0.88	Number of patients in %:** TAM**: N1: 7.4, P1: 20.2, P2: 62.8, DY: 9.6; **controls**: N1: 19.7, P1: 38.3, P2: 37.2, DY: 4.8

Hong and Ki 1999 [37]	Effect of tamoxifen on breast density in women who had undergone surgery for BC	Retrospective case control study	BC	152 BC patients/172 (**group I**: 102 BC patients (**TAM** only: 40; +chemotherapy: 36; +radiotherapy: 13; +radiotherapy and chemotherapy: 13), **group II**: 50 BC patients without TAM, **group III**: 20 healthy women)	Mean age: **group I**: 49 y (range 28–67 y); **group II**: 47 y (range 32–58 y); and **group III**: 47 y (range 34–62 y)	Visually according to a 3-score classification; decrease of BD: **0** = <10%, **1** = 10–20%, **2** = 20%/at baseline: **BC patients**: before and after surgery and for **control group**: once a year for at least 2 years, and on follow-up	Two readers	NA	Number of patients^4^: dense pattern: **group I**: 19 (18.63%), **II**: 13 (26%),** III**: 7 (35%). Heterogeneously dense: **group I**: 44 (43.13%), **II**: 21 (42%), **III:** 9 (45%). Fatty pattern: **group I**: 39 (38.24%), **II**: 16 (32%), **III**: 4 (20%)

Konez et al. 2001 [38]	Effect of tamoxifen on breast density	Retrospective cohort study	BC	27 BC patients without chemotherapy/24 BC patients	Mean 67 y (32–81 y)	Visually and using densitometric values (ratio dense/fat)/at baseline, within 2 or 3 yrs, at 5 yrs and 1 yr after cessation of treatment	Two radiologists blinded to the time sequence of the mammograms	Agreement in 24/27 cases^5^	Mean values of the relative glandular densities (glandular tissue/fat density) at baseline: 0.652

Meggiorini et al. 2008 [39]	Effect of tamoxifen on breast density	Retrospective case control study	BC	148 BC patients/**cases**: 68 BC patients who receive tamoxifen alone or ± radiotherapy and chemotherapy; **controls**: 80 BC patients ± radiotherapy and chemotherapy	**TAM group**: mean 58.5 ± 9.3 y, median 56.5 y, range 41–78 y; **controls group**: mean 63.9 ± 9.2 y, median 63.5 y, range 49–78 y	Visually according to BIRADs and by a computer-assisted method after digitizing images/at baseline and annually for six years	Two radiologists blinded to the treatment	Agreement between methods for baseline mammograms: cases = 0.994 *P* < 0.00001, controls = 0.985 *P* < 0.00001 and mammograms at 1 yr: cases = 1, controls = 1	Basal mammography with classification BIRADS (number of patients): in **TAM group**: A: 22, B: 14, C: 22, D: 10 and **control group**: A: 30, B: 12, C: 26, D: 12

Chen et al. 2011 [40]	Effect of tamoxifen on breast density	Retrospective cohort study	BC	17 BC patients without chemotherapy/16 BC patients treated by **TAM** after surgery and without chemotherapy	Mean 43 y, range 33–51 y	Based on 3D MRI T1-weighted images. The changes in breast volume (ΔBV), fibroglandular tissue volume (ΔFV), and percent density (Δ%BD) between 2 MRI studies were analyzed/pretreatment and follow-up studies ranged from 8 to 26 months (17.5 ± 5.7 months)	NA	NA	Baseline %BD ranged from 5.1% to 39.5% (22.1 ± 2.6%)

Cuzick et al. 2011 [41]	Effect of tamoxifen-induced reductions in breast density on BC risk	Nested case control study within a randomized prevention trial (IBIS 1)	At least twice the average BC risk of a 50-year-old woman: either benign proliferative breast disease or a strong family history of BC^6^	1288 women (potential control subjects without BC: *n* = 1064; potential case subjects with BC: *n* = 224)/1065 women **with mammograms at baseline and follow-up **(12–18 months after trial entry). **Cases**: 123 women diagnosed with BC; **control subjects**: 942 women without BC	Mean 51 ± 6 y, range 35–70 y	Visual assessment of the proportion of the total breast area that was composed of dense tissue (to the nearest 5%)/at baseline (at or up to 12 months before randomization), and at first follow-up (18–23 months)	One radiologist blinded to treatment arm and not case control status	Reproducibility assessed for 48 women by 5 readers at baseline and follow-up (Pearson correlation coefficient *r* ranged from 0.48 to 0.98)	**Case subjects**: mean MD at entry: 51%; **control subjects**: 44% (***P*** = 0.02)

BC: breast cancer. ^1^Low-dose tamoxifen of 5 mg. ^2^IEN: intraepithelial neoplasia including ductal and lobular carcinoma in situ; T1: pT1 a or pT1mic N0. ^3^For 40 women BD was not measured at baseline. DB with digital measurement: 16% lower compared to those with analog film screen. DCIS: ductal carcinoma in situ. LCIS: lobular carcinoma in situ. NA: nonavailable or not applicable; SD: standard deviation. Wolfe classification: N1: nondense, no ducts visible; P1: prominent ductal pattern occupying less than one-fourth of the breast; P2: prominent ductal pattern occupying more than one-fourth of the breast; DY: homogenous, plaque-like areas of density. ^4^Personal calculation for percentages. ^5^For 24 cases: results revealed parallelism in the findings of the two radiologists. MD: mammographic density. BI-RADS breast categories: (1) almost entirely fat, (2) fatty with scattered fibroglandular densities, (3) heterogeneously dense breast tissue, and (4) extremely dense breast tissue. ^6^i.e., a mother or sister who developed BC before the age of 50.

**Table 2:** Raloxifene (RLX): characteristics of the reviewed studies (*n* = 9).

Authors/year	Endpoint	Design	High risk definition	Population/number of cases	Age	Method of breast density measurement/time	Mammograms readers	Reproducibility	BD baseline
Jackson et al. 2003 [42]	Effect of raloxifene and HRT on breast density	Randomized, open-label, parallel designed trial	NA	356 postmenopausal women with osteopenia or osteoporosis/280 assigned randomly/193 included in the final analysis: **RLX**: 109; **ccHT**: 84^1^	Mean 66.7 y; average y postmenopause: 17.9; **RLX** (mean ± SD): 66.9 y ± 5.3; ccHT: 66.4 y ± 4.5	Visually according to BI-RADs classification at baseline and after 12 months of therapy	Three radiologists blinded to treatment assignment	Weighted Kappa for interrater reliability at baseline: 0.57 to 0.70; after 12 months: 0.51 to 0.66	BI-RADs I: 16.6–22.8%; II: 65.3–75.6%; III: 7.8–10.9%; IV: 0-1%

Eilertsen et al. 2008 [43]	Effect of raloxifene and different regimens of HT on breast density	Open-label, randomized, comparative clinical trial	NA	358 postmenopausal women/202 assigned randomly/178 included in the final analysis: **low-dose combined HT**: *n* = 44, **conventional-dose combined HT**: *n* = 45, **tibolone**: *n* = 45, and **RLX**: *n* = 44^2^	45–65 yrs:** low-dose combined HT (**mean ± SD): 54.8 y ± 5.0; **conventional-dose combined HT**: 56.1 y ± 3.6; **tibolone**: 54.9 y ± 4.7; **RLX**: 56.3 y ± 4.9	Volumetric breast density by a fully automated technique in full-field digital mammograms at baseline and after 12 weeks	NA	NA	**Low-dose HT**: 8.6 (5.1–17.1); **conventional-dose HT**: 8.3 (5.5–11.4); **tibolone**: 7.5 (5.3–12.3); **RLX**: 7.7 (5.7–11.1)^3^

Eng-Wong et al. 2008 [44]	Effect of raloxifene on breast density	Retrospective analysis of a phase II prospective trial	Gail 5-year risk ≥ 1.7%, or a family history of BC, or previous LCIS, ADH, DCIS	37 high risk premenopausal	43 y (35–47)	On digitized mammograms using a semiquantitative technique and using MRI T1-weighted images to determine breast **MRI volume** using a semiautomatic method at baseline, after 1 and 2 years, and 1 year posttreatment	Two radiologists	Previously tested in another validation study for MRIV (94.95% agreement). For mammograms: interradiologist correlation: 1 y: 0.63; 2 y: 0.62; 3 y (one year off **RLX**): 0.39	Mean BD (range) 39% (7–78)

Freedman et al. 2001 [45]	Effect of raloxifene (at one of two doses) and HRT on breast density	Subgroup analysis of a prospective double-blind, randomized, placebo-controlled trial	NA	619 postmenopausal women with previous hysterectomy/168 included in the final analysis: **placebo** (*n* = 45); **RLX** 60 mg/day (*n* = 45); 150 mg/day (*n* = 42); **estrogen** 0.625 mg/day (*n* = 36)	Mean 53 y (45–60); mean y after menopause: 6. **Placebo** (mean ± SD): 52.2 y ± 5.1; **RLX** 60 mg/day: 54.1 y ± 4.2; 150 mg/day: 53.3 y ± 4.8; **estrogen**: 52.1 y ± 4.6	Digital scanning and computer-assisted segmentation of mammograms at baseline and 2 years	One radiologist blinded to treatment	Previously tested in another validation study	**Placebo**: 9.8% ± 9.6%; **RLX** 60 mg/day: 9.3% ± 9.1%; 150 mg/day: 8.1% ± 6.6%; **estrogen**: 13.5% ± 11.5%

Harvey et al. 2009 [46]	Effect of bazedoxifene compared with raloxifene or placebo on breast density	Retrospective analysis of a phase III randomized placebo- and active-controlled trial	NA	7 609 postmenopausal women with osteoporosis/1 243 eligible for participation; 622 eligible for digitization and 442 included in the final analysis: **bazedoxifene** 20 mg (*n* = 92); **bazedoxifene** 40 mg (*n* = 106); **RLX **60 mg (*n* = 119); **placebo **(*n* = 125)	≤62 y; mean age: 58.7 y. Mean ± SD: **bazedoxifene** 20 mg/day: 58.3 y ± 2.5; 40 mg/day: 58.8 y ± 2.4; **RLX** 60 mg/day: 58.8 y ± 2.6; **placebo**: 58.8 y ± 2.5	On digitized mammograms using an interactive thresholding software at baseline and at 2 years	One radiologist blinded to time sequence and treatment arm	NA	**Bazedoxifene** 20 mg: 26.4% (SD 18.7%); **bazedoxifene **40 mg: 25.8% (SD 19.1%); **RLX** 60 mg: 27.6% (SD 19.3%); **placebo**: 27.2% (SD 18.1%)

Nielsen et al. 2009 [47]	Effect of transdermal estradiol compared with raloxifene on breast density or heterogeneity	Post hoc analysis of a prospective randomized study	NA	500 women at least 5 y postmenopausal/270 included in the final analysis: **RLX** 60 mg/day (*n* = 135); **low-dose estradiol** (*n* = 135)^4^	66 years (55–80) mean ± SD: **RLX**: 66.7 y ± 0.5; **estradiol**: 66.3 y ± 0.5	Visually according to BI-RADs classification, area percentage and computer-based (E2-specific) heterogeneity examination of digitized mammograms at baseline and at 2 years	One radiologist blinded to treatment	Previously tested in another validation study: intraobserver variability between baseline and 2-year assessments = 0.79 (range 0.70–0.86)	**RLX** 60 mg: 16% (5–31); **low-dose estradiol**: 16% (7–24)

Lasco et al. 2006 [48]	Effects of long-term raloxifene on breast density	Prospective case control study	NA	70 postmenopausal women with normal body weight/cases: women with osteoporosis receiving **RLX** 60 mg/day (*n* = 50); **controls**: women without osteoporosis (*n* = 20)	**Cases**: 52.4 ± 4.1 y, menopausal age: 42.1 ± 3.9 y; **controls**: 53.6 ± 3.5 y, menopausal age: 43.1 ± 3.6 y	Digital scanning and computer-assisted segmentation of mammograms at baseline and 2 years	NA	NA	Image mean index (IMI)^5^: ~3.35 for both groups

Christodoulakos et al. 2002 [49]	Effect of raloxifene compared with tibolone on breast density	Randomized, comparative clinical trial	NA	131 postmenopausal women/**tibolone** 2.5 mg/day (*n* = 56), **RLX** 60 mg/day (*n* = 48) and controls: no risk factors of osteoporosis or denied treatment: (*n* = 27)	Mean age ± SD: **tibolone**: 52.6 y ± 4.8, **RLX**: 53.9 y ± 3.9, controls: 51.4 y ± 7.6, mean months since menopause: range 69.8–88.3	Visually according to Wolfe ^6^classification at baseline and 12 months	Two radiologists blinded to treatment arm	In case of discrepancies (9.2–13%), films were reevaluated by the 2 radiologists together for consensus	**N1**: tibolone: 19 (36.5%), RLX: 23 (47.9%), controls: 6 (22.2%); **P1**: tibolone: 16 (30.8%), RLX: 10 (20.8%), controls: 7 (25.9%); **P2**: tibolone: 15 (28.8%), RLX: 12 (25%), controls: 11 (40.7%); **DY**: tibolone: 2 (3.8%), RLX: 3 (6.3%), controls: 3 (11.1%)^7^

Cirpan et al. 2006 [50]	Effect of raloxifene on breast density	Retrospective study	NA	55 postmenopausal women with osteopenia or osteoporosis	Mean age ± SD: 50.4 y ± 3.6 (43–58)	Visually according to BI-RADs classification at baseline and after 12 to 16 months of therapy	NA	NA	BI-RADs category **I**: *n* = 8 (14.5%);** II**: *n* = 28 (50.9%);** III**: *n* = 17 (30.9%);** IV**: *n* = 2 (3.6%)

^1^ccHT: continuous-combined HT: conjugated equine estrogen 0.625 mg/day + medroxyprogesterone acetate 2.5 mg/day. ^2^Conventional-dose HT = 2 mg 17 *β*-estradiol and 1 mg norethisterone acetate, low-dose HT = 1 mg 17 *β*-estradiol and 0.5 mg norethisterone acetate. NA: not available or not applicable. ^3^Values are represented as median (25th–75th percentile of interquartile range). LCIS: lobular carcinoma in situ, ADH: atypical ductal hyperplasia, and DCIS: ductal carcinoma in situ. ^4^Weekly patch delivering 0.014 mg estradiol/day. ^5^Image Mean Index (IMI): computer-assisted algorithm calculation including identification and delimitation of the same region of interest for each image and the differentiation of gray levels into 10 classes. ^6^Wolfe classification: N1: parenchymal pattern composed almost entirely of fat with trabeculae but no visible ducts, P1: pattern composed mainly of fat with fibroglandular tissue that constitutes 25% of the breast, P2: pattern composed of fibroglandular tissue appearing as a heterogeneously dense breast that occupies more than 25% of the breast, and DY: extremely dense tissue. ^7^Tibolone versus controls: *P* = 0.32 or RLX versus controls: *P* = 0.18.

**Table 3:** Aromatase inhibitors: characteristics of the reviewed studies (*n*: 7).

Authors/year	Endpoint	Design	High risk definition	Population/number of cases	Age	Method of BD measurement/time	Mammograms readers	Reproducibility	BD baseline
Letrozole (LET)									

Cigler et al. 2010 [51]	Effect of letrozole on breast density	A multicenter, randomized, double-blind, placebo-controlled trial (2 : 1 ratio of **LET** (2.5 mg daily) or placebo)	NA	Healthy postmenopausal women with or without prior BC and with an estimated >25% BD on baseline mammogram/a total of 120 patients would be required; 67 women were randomized and 50 were included in the final analysis (**LET**: 31, **placebo**: 19)^1^	Median: 57.4 y; **LET**: 57.3 y; **placebo**: 58.1 y	Visually according to Boyd classification and BIRADs systems at baseline and 12 months and by a computer-assisted thresholding program at baseline, 12 and 24 months	A single observer blinded^1a^	NA	Mean baseline BD: 39.6% (95% CI 32.3–47.0) for women on **LET** and 40.0% (95% CI 32.4–47.7) on **placebo**

Vachon et al. 2007 [52]	Effect of letrozole on breast density	Subset analysis of a prospective randomized trial	NA	Women with early-onset BC after 5 yrs of tamoxifen/204 women were potentially eligible; 104 women met all study criteria: **LET**: *n* = 56 (54%); **placebo**: *n* = 48 (46%)	Median age (range): **placebo**: 64.7 y (49–80); **LET**: 64.6 y (30–84)	On digitized mammograms using a computer-assisted thresholding program at baseline and 9–15 months	A trained programmer	Intraobserver variation no greater than 10% on over 500 images	**Placebo/LET** 0–9%: 12 (26%)/15 (28%); 10–24%: 20 (43%)/21 (39%); 25–49%: 12 (26%)/15 (28%); 50–74%: 3 (6%)/3 (6%); 75+%: 0 (0%)/0 (0%). Mean (SD): **placebo** (*n* = 33): 20% (14.1); **LET** (*n* = 35): 18.5% (14.5)

Smith et al. 2012 [53]	Effect of letrozole on breast density	Prospective, single-arm study	LCIS, ADH, ALH, or DCIS, or BRCA-1 mutation or a Gail 5 y of BC > 8%	Postmenopausal women with risk factors for developing BC/20 enrolled, 16 evaluable	Median: 58.9 y	On digitized mammograms using a computer-assisted thresholding software at baseline, 6 and 12 months	An experienced reader blinded to time sequence	NA	Range: 1.049% to 64.1425% (median: 27.70%)^2^

Fabian et al. 2007 [54]	Effect of letrozole on biomarkers of BC risk including breast density	Phase II biomarker based pilot prospective, single-arm study	(1) Gail 5 y of BC ≥ 1.67%; (2) prior ADH, ALH, or LCIS; (3) prior treated contralateral DCIS; (4) known BRCA1/2 mutation	Postmenopausal women with risk factors for developing BC and who were on HRT/42 women^3^	Median 50 y (range 39–68)	On digitized mammograms using a computer-assisted thresholding software at baseline and 6 months	A single reviewer blinded to the time sequence of mammograms	NA	Median BD at baseline for the entire group: 31.5%

Mousa et al. 2008 [55]	Effect of letrozole on breast density	Preliminary retrospective case control study	NA	Postmenopausal women who were on HRT/56 women: cases (*n* = 28) received low-dose HRT daily + LET 2.5 mg and controls: HRT alone (*n* = 28). BD assessed for 18 women in cases and 22 in controls^4^	For 18 cases and 22 controls: mean ± SD: cases: 63.06 y ± 6.99; controls: 65.45 y ± 8.28	Visually according to BI-RADs system and by a computer-assisted thresholding program at initial time and after 24 months of treatment	One radiologist blinded to time sequence and treatment arm	Correlation coefficient for the intraobserver reliability: 0.92 for the visual scores of BD	Total IPI for 1st mammogram: mean ± SD: cases (*n* = 18): 2.1 × 10^10^ ± 2.6 × 10^10^; controls (*n* = 22): 1.5 × 10^10^ ± 2.4 × 10^10^

Anastrozole (ANAS)									

Prowell et al. 2011 [56]	Effect of anastrozole on breast density	Prospective, single-arm study	NA	Postmenopausal women with BC with aromatase inhibitor^5^as their only systemic therapy/54 patients; 50 assessed for breast density measurement, 43 patients had both baseline and 12-month mammograms	Mean age (range): 62.5 y (47–79)	On digitized mammograms using a computer-assisted thresholding program at baseline, 6 months and 12 months	A single observer blinded to time sequence	NA	Median BD at baseline (range): 13.4% (0.1–66.2)

Exemestane (EXE)									

Cigler et al. 2011 [57]	Effect of exemestane on breast density	Prospective double-blind randomized trial (1 : 1 ratio)	NA	Healthy postmenopausal women/98 women were randomized (49 to **EXE** 25 mg; 49 to **PLAC**) and 65 had BD data at baseline and 12 months, and 43 at baseline and 24 months	Median: 56.9 y median**: EXE**: 56.9 y**, Placebo**: 56.8 y	Visually according to Boyd classification and BI-RADs system at baseline and on digitized mammograms using a computer-assisted method at baseline, 6, 12, and 24 months	A trained physician blinded^1a^	NA	Mean baseline BD: 33.9% (95% CI from 27.6 to 40.2) for women on **EXE** (*n* = 38) and 36.5% (95% CI from 33.3 to 42.8) for women on **placebo** (*n* = 34)

^1^Only 43 women (27 on letrozole and 16 on placebo) had both baseline and 24 months. ^1a^Blinded to any clinical data corresponding to each mammogram. LCIS: lobular carcinoma in situ, ADH: atypical ductal hyperplasia, ALH: atypical lobular hyperplasia, and DCIS: ductal carcinoma in situ. ^2^Personal calculation. HRT: hormonal replacement therapy. ^3^Thirty-five of 42 subjects were taking estrogen alone (various preparations) and seven estrogen + progesterone. Fifty-nine percent were on transdermal preparations. ^4^Study participants: all received letrozole (Femara) 2.5 mg orally 3 times weekly with the exception of 2 women who were given anastrozole (Arimidex) 1 mg/day because of headaches with letrozole. IPI: computerized calculation of integrated pixel intensity. ^5^Anastrozole 1 mg daily.

**Table 4:** Tamoxifen (TAM) studies: results: effect on BD and confounding factors of treatment interaction.

Authors	Change in BD			Confounding factors	
	Treatment	Endpoint	*P *	At baseline	Interaction with treatment
Cuzick et al. 2004 [32]	Tam PLAC	*Reduction in % density at 54 months* −13.7% (95% CI −12.3% to 15.1%) −7.3% (95% CI −6.1% to 8.4%)	0.001 0.001	Statistically significant higher BD at baseline was associated with low BMI, age ≤ 55 y, no familial risk of BC, past breast biopsy, and never smoking	In **MVA**, greater reductions in BD were associated with treatment arm (Tam), BMI <25 kg/m^2^ or less, stopping HRT during the study, smoking during the study, and higher BD at entry. Treatment effect statistically smaller in older and postmenopausal women^1^

Decensi et al. 2009 [33]		*Reduction in % density at 24 months* ^ 2^		Statistically higher BD at baseline was associated with low BMI, younger age (details NA)	In **MVA, BD** at entry had no statistically significant interaction with treatment effect
	Tam	−19.6% (95% CI −26.3% to −12.9%)	0.003		
	Fenretinide	−10.8% (95% CI −23.8% to 2.1%)			
	Both	−21.6% (95% CI −29.0% to −14.3%)			
	Placebo	−12.2% (95% CI −25.2% to 0.9%)			

Chow et al. 2000 [34]		*Reduction in % density per year* ^ 3^		No statistically significant effect of age on BD	In **MVA**, age and menopausal status had no statistically significant interaction with treatment effect (***P* = NA**)^4^
	Tam	−4.3%	0.0007		
	Control	No change			

Brisson et al. 2000 [35]		*Reduction in % density ± SD posttreatment *		No statistically significant effect of age on BD	In **MVA**, age, nulliparity, and history of breast biopsy had no statistically significant interaction with treatment effect
	Tam	−9.4% ± 12.0	0.010		
	Plac	−3.6% ± 5.3			

Atkinson et al. 1999 [36]		*Women with decreased BD: n (%) *		**NA**	**N** **M** **T** **A** ^5^
	Tam	29 (33%)	0.0001		
	Control	10 (5%)			

Hong and Ki. 1999 [37]		*Women with decreased BD: n (%) *		**NA**	In **MVA**, menopausal status had an interaction with treatment effect: decrease in BD (*n*): premenopause: 47/54 (87%); postmenopause: 14/48 (29.2%). (***P* = NA**)
	Tam	61 *(59.8%) *	**NA**		
	Group II	18 (36%)			
	Group IIIl	2 (10%)			

Konez et al. 2001 [38]		*Women with decreased BD: n (%) *		**NA**	**NMTA**
	Tam	5/24 (21%) (within treatment: *n*: 3 and at end *n*: 2)			
		*Relative glandular density change per interval*: 0.012 ± 0.006	0.06^6^		

Meggiorini et al. 2008 [39]		*Women with decreased BD: n (%) *		**NA**	In **MVA**, greater reduction of BD was statistically significantly associated with age <59 y (***P* = 0.035**)
	Tam	14/68 (20.59%)	0.021		
	Control	6/80 (7.50%)			

Chen et al. 2011 [40]	Tam	*Absolute reduction of %BD (Δ%BD)* ^ 7^ −5.8% ± 3.8% (95% CI: −3.7% to −7.8%)	0.001	**NA**	**NMTA**
Cuzick et al. 2011 [41]		*Reduction ≥ 10% in % density *		Statistically significant higher BD at baseline in women with atypical hyperplasia or LCIS	In **MVA**, atypical hyperplasia and LCIS had no significant interaction with treatment effect (***P* = 0.4**), greater reduction in BD in women who had never taken HRT **(*P* = 0.03)** ^9^
	*All patients* (*N* in %)				
	Tam	232/507 (45.7%)			
	Placebo	140/558 (25%^8^)			
	Control subjects in Tam group	48%	<0.001		
	Control subjects in Plac group	26%			
	Case subjects in Tam group	29%			
	Control subjects in Tam group	48%	0.01		

^1^Using additional Student's *t*-tests to determine whether the observed effects of tam on BD applied equally to all subgroups of women; in women ≤45 yr at entry, the net reduction with tam: 13.4% (95% CI −8.6% to 18.1%), whereas in women >55 y: 1.1% (95% CI −3.0% to 5.1%). MVA: multivariable analysis. ^2^Using analog mammograms only. ^3^Using digitized mammograms: SD ± 6.6%, range = −21.5 to 10.1. ^4^Because of the small numbers of patients in these groups, the power to detect mean differences (6–8% between them) is low. NMTA: no multivariable analysis of interaction with treatment effect available. ^5^There was too much missing data in order to assess the effect of previous use of HRT on change in BD. NA: data non available. ^6^Calculated in 27 cases. ^7^Evaluated by 3D MRI (magnetic resonance imaging). ^8^Personal calculation for percentages. LCIS: lobular carcinoma in situ. ^9^After adjusting for age and BD at IBIS-I entry, body mass index at IBIS-I entry, age at menarche, menopausal status at entry to IBIS-I, smoking, family history, benign breast disease (atypical hyperplasia or LCIS), and treatment arm, the mean reduction in breast density was smaller in women who started HRT during the study compared with those who had never taken HRT (4.3% versus 6.3%), mean difference in breast density reduction = 2.02%, 95% CI = 0.02% to 3.83%, *P* = 0.03.

**Table 5:** Raloxifene (RLX ***n*: 9**) and aromatase inhibitors (Let, ANAS, and EXE ***n*: 7**) studies: results: effect on BD and confounding factors of interaction.

Authors	Change in BD					Confounding factors
	Treatment	Endpoint			*P *	
Jackson et al. 2003 [42]		*Increased BD, patients number (%) *				
	**RLX**	1 (0.9%)			<0.001	**NMTA**
	**ccHRT**	23 (27.4%)				

Eilertsen et al. 2008 [43]		*Median changes in volumetric BD % *				
	(I) Low-dose HT	+17.2% (95% CI 1.1–28.7)			0.001	
	(II) Conventional HT	+15.0% (95% CI 4.8–28.6)			0.001	**NMTA**
	(III) Tibolone	+0.7% (95% CI 9.2-to 7.3)			0.9	
	(IV) Rlx	–4.1% (95% CI 6.9 to 2.1)			0.09	

Eng-Wong et al. 2008 [44]		*Median % Δ MRIV *				Mean BMI (range) 24 (18–41) and mean age (range) 43 y (35–47) are not significantly correlated with BD at baseline, NMTA
	1 year	**−17%** (95% CI −28 to −9)			0.0017	
	2 years	**−16%** (95% CI −31 to −14)			0.0004	
	3 years	**−9% **(95% CI −18 to 20)			0.64	

Freedman et al. 2001 [45]		*Mean change in BD % *				No statistically significant therapy-by-subgroup interactions (for BMI, age at entry, menopausal status, use of HRT, baseline breast density, and smoking status)
	Placebo	−1.3% ± 2.9%				
	RLX 60 mg	−1.5% ± 4.1%			<0.02^1^	
	RLX 150 mg	−1.7% ± 3.4%				
	Estrogen	+1.2% ± 5.3%				

Harvey et al. 2009 [46]		*Mean change in BD %(SD) *				
	Bazedoxifene 20 mg	−1.2% (4.4)				
	Bazedoxifene 40 mg	−0.4% (3.5)			NS	**NMTA**
	RLX 60 mg	−0.5% (3.4)				
	Placebo	−0.2% (3.0)				

Nielsen et al. 2009 [47]		*Area % *	*E2-HER* ^ 2^			BMI: negatively correlated with BD at baseline **NMTA**
	RLX	+2%^3^	+0.41		<0.05 for area
	Estradiol	+4%	+0.32		<0.0001 for^2^

Lasco et al. [48]		*Image mean index (%) *				
	RLX	−1.9%			<0.05	**NMTA**
	Controls	No significant change				

Christodoulakos et al. 2002 [49]	*Patients number (%) *	*No change *	*Increase *	*Decrease *		**NMTA**
	RLX	36 (75)	3 (6.3)	9 (18)	**NA**	
	Tibolone	44 (78.6)	6 (10.7)	6 (10.7)		
	Controls	20 (74.1)	—	7 (25.9)		

Cirpan et al. 2006 [50]	RLX	No change in BIRADs categories^4^			=0.32	**NMTA**

Cigler et al. 2010 [51]		*Mean change *				No statistically significant therapy-by-subgroup interactions (for BMI and age at entry)
	Letrozole	**−0.01% ** (95% CI −3.89 to 3.87)			=0.69	
	Placebo	**−1.32% **(95% CI −8.86 to 6.22)				

Vachon et al. 2007 [52]		*Mean change *				No statistically significant therapy-by-subgroup interactions (for BMI and age at entry)
	Letrozole	−2.7%			=0.96	
	Placebo	−3.0%				

Smith et al. 2012 [53]	*Patients number *	*Decrease *	*Increase *		0.0358	**NMTA**
	Letrozole	11/16	3/16			

Fabian et al. 2007 [54]		*Mean relative change *			**NA**	**NMTA**
	Letrozole + HRT^5^	0.4 (Baseline: 31.5 %; Posttreatment: 29.4 %)				

Mousa et al. 2008 [55]		* Δ % dense IPI (SD)* ^ 6^				**NMTA**
	Letrozole + HRT	−6.84 ± 9.0			0.044
	HRT alone	−1.41 ± 8.17			

Prowell et al. 2011 [56]	Anastrozole	*Mean change * −**16%** (95% CI: −30 to 2)			0.08	No statistically significant therapy-by-subgroup interactions (for BMI and age at entry)

Cigler et al. 2011 [57]		*Mean change *				No statistically significant therapy-by-subgroup interactions (for BMI and age at entry)
	Exemestane	−0.17 (95% CI from −4.34 to 4.00)			0.37	
	Placebo	−2.93 (95% CI from −8.70 to 2.85)				

ccHRT: continuous-combined hormone therapy that consisted of conjugated equine estrogen 0.625 mg/day plus medroxyprogesterone acetate 2.5 mg/day. NMTA: no multivariable analysis of interaction with treatment effect available. *Δ*MRIV = change in breast MRI volume. ^1^The mean density within the placebo group and both raloxifene groups decreased statistically significantly from baseline (*P* < 0.02 for each group), while the mean density in the ERT (estrogen replacement therapy) group increased but not statistically significantly. ^2^E2-HER: computer-based (E2-specific) heterogeneity examination of radiographs. ^3^None of the patients in either treatment group demonstrated a decrease in breast density; using area % measurement: no significant increase from baseline. NA: not available. ^4^Only in one patient did the BI-RADS classification of 2 change to 3 after 12 months of therapy. ^5^HRT: hormonal replacement therapy. ^6^IPI: computerized calculation of integrated pixel intensity.
